# New approaches to the study of sepsis

**DOI:** 10.1002/emmm.201201375

**Published:** 2012-12-04

**Authors:** Peter A Ward

**Affiliations:** Department of Pathology, University of Michigan Medical SchoolAnn Arbor, MI, USA

**Keywords:** clinical trials, complement, interventions, mediators, sepsis

## Abstract

Models of sepsis have been instructive in understanding the sequence of events in animals and, to an extent, in humans with sepsis. Events developing early in sepsis suggest that a hyperinflammatory state exists, accompanied by a buildup of oxidants in tissues reflective of a redox imbalance. Development of immunosuppression and degraded innate and adaptive immune responses are well-established complications of sepsis. In addition, there is robust activation of the complement system, which contributes to the harmful effects of sepsis. These events appear to be associated with development of multiorgan failure. The relevance of animal models of sepsis to human sepsis and the failure of human clinical trials are discussed, together with suggestions as to how clinical trial design might be improved.

## Introduction

**Figure 1:**
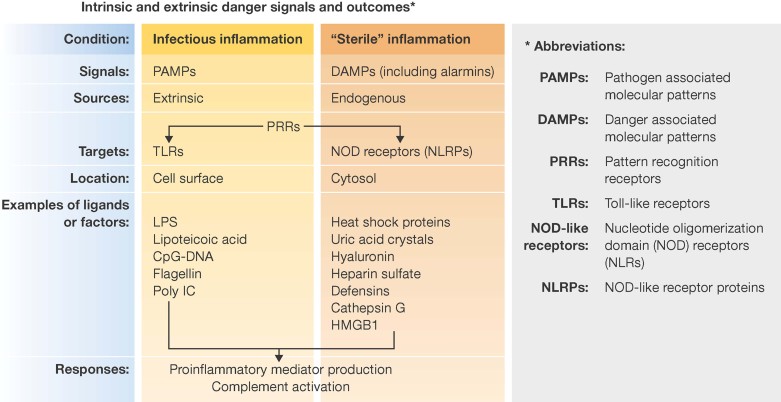
Intrinsic (DAMPs) and extrinsic (PAMPs) signals develop during an infectious condition (*e.g.* bacterial pneumonia) that causes inflammation and sepsis which is often associated with development of SIRS, buildup of ROS and RNS in tissues, multiorgan failure (MOF) and lethality Receptors (PRRs) for these signals engage both TLRs and NOD-like receptors. The listings of ligands that interact with TLRs and NOD receptors is somewhat artificial. For instance, while HMGB1 (considered to be a DAMP) interacts with TLR4, it also interacts with TLR2 and with the receptor for advanced glycation products (RAGE). Heat shock proteins (DAMPs) react with TLR2, TLR4 and with receptors on antigen presenting cells (CD36, a scavenger receptors). ‘Sterile’ inflammation occurs after hemorrhagic shock, polytrauma, ischemia/reperfusion and is not usually associated with the presence of an infectious agent. In all cases, the same cascade of downstream events seems to occur.

Sepsis is a clinical condition that was originally assumed to be a systemic response to bacterial infection, but it is now clear that other infectious agents (*e.g.* viral, fungal and parasitic organisms) can also trigger sepsis. Sepsis can develop secondary to release of various bacterial components [*e.g.* lipopolysaccharide (LPS) from Gram negative bacteria, lipoteichoic acid from Gram positive bacteria] that interact with toll-like receptors (TLRs) to trigger inflammatory responses. More recently, it has been discovered in cases of ‘sterile infection’ that a sepsis-like condition can also develop (Chen & Nunez, 2010). Examples of ‘sterile infection’ resulting in sepsis-like responses include severe non-penetrating polytrauma (such as multiple bone fractures and soft tissue injury), ischemia-perfusion injury and haemorrhagic shock. In such cases, the TLR system is also activated. In bacterial sepsis, the agonists for TLRs are referred to as pathogen-associated molecular patterns (PAMPs; Bianchi, 2007; Zipfel & Robatzek, 2010; Fig 1). PAMPs are exogenous signals usually derived from infectious agents and are interactive with pattern recognition receptors (PRRs) including TLRs (present on cell surfaces and intracellularly) and NOD receptors (present in the cytosol) involving numerous cell types. Products released in ‘sterile sepsis’ are referred to as danger-associated molecular patterns (DAMPs) that can trigger inflammatory responses often via interaction with TLRs. DAMPs include endogenous danger signals such as DNA, histones, heat shock proteins, hyaluronins and heparin sulphate released from damaged or necrotic cells and other products (Fig 1). A subset of DAMPs are the ‘alarmins’ that were recently described (Bianchi, 2007; Oppenheim et al, 2007; Yang et al, 2009) and include cell constituents such as granulolysins, defensins, lactoferrin, cathepsin G, HMGB1, urate crystals, ATP, etc. Some DAMPs are enzymes (*e.g.* ATPases). Other DAMPs, such as HMGB1, are peptides reactive with TLRs and other receptors. When DAMPs appear extracellularly, they react with cell surface receptors or with other proteins or substrates (*e.g.* ATPases) to trigger inflammatory responses. Intracellular TLRs (3,7,9) react with double or single stranded RNA. DAMPs have also been shown to play roles in inflammatory responses following ischemia/reperfusion injury in the heart, kidneys, liver and lungs (Pardo et al, 2008). Collectively, sufficient amounts of DAMPs can trigger a sepsis-like response resulting in a ‘cytokine storm’ [defined as presence of proinflammatory cytokines/chemokines in plasma and also referred to as the systemic inflammatory response syndrome (SIRS)].

In spite of a great deal of investment of time and money in basic and clinical research in sepsis, including more than 40 clinical trials in septic humans, it is disconcerting that there is no FDA-approved drug for use in sepsis. Recently, Xygris (recombinant activated protein C) and Eritoran (an inhibitor of TLR4) were withdrawn because of lack of clinical efficacy in sepsis (Angus, 2011). This has caused great consternation in the investigative community and has resulted in large pharmaceutical companies being very risk-adverse for investing in drug development and clinical trials in sepsis. It is not entirely clear why there has been such dismal failure. Part of the problem may be the relevance of animal models as surrogates of human sepsis. Some of the difficulty may also be in clinical trial design, both of which are described in this review.

Sepsis in humans is linked to the presence of an infectious organism in approximately 50% of cases. This calculation is probably an underestimate due to the fact that by the time patients have been admitted to the intensive care unit (ICU), they are often on ventilator support and on vasopressors to maintain adequate blood pressure and often have already been treated with broad spectrum antibiotics before admission to the ICU, complicating the ability to identify a causative organism. Clinically, sepsis has been classified as: sepsis, severe sepsis (with SIRS), followed by the presence of multiorgan dysfunction (MOF), and septic shock (Tang et al, 2010). Progression of sepsis may be due to the inability to contain infectious agents, an example being a leak at a surgical anastomotic site in the colon. Sepsis can also progress because of release of PAMPs or DAMPs. Whatever triggered development of sepsis, the ensuing result is development of SIRS, together with tissue buildup of reactive oxygen species [ROS; including superoxide anion (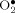), H_2_O_2_, and myeloperoxidase products of H_2_O_2_, and the hydroxyl radical (HO^**.**^)]. Reactive nitrogen species [RNS; such as NO^**.**^ (nitric oxide) and peroxynitrite anion (ONOO^**.**^)] are also produced. ROS and RNS are reactive with proteins, lipids and DNA, forming adducts. ROS can eventually form a variety of products such as exocyclic ethano-DNA adducts with deoxyadenosine or deoxycytidine (Fang, 2004). In the case of DNA, this can ultimately lead to DNA strand breaks, which then activates the repair enzyme, polyadenosine ribose polymerase (PARP). PARP activation can cause substantial depletion of mitochondrial ATP (Angus, 2010) resulting in defective mitochondrial transfer of electrons and contributing to the buildup of ROS.

**Glossary**

**Cecal ligation and puncture (CLP)**

Widely used experimental model for sepsis, in which sepsis originates from a polymicrobial infectious focus within the abdominal cavity, followed by bacterial translocation into the blood compartment, which then triggers a systemic inflammatory response.

**Chemokines**

A family of small cytokines, which induce directed chemotaxis in nearby responsive cells (**chemo**tactic cyto**kines**).

**Complement**

Part of the innate immune system; a group of proteins present in blood plasma and tissue fluid, which combine with an antigen–antibody complex to induce the lysis of foreign cells.

**Cytokines**

Intercellular protein mediators released by immune cells to regulate the immune response.

**Danger-associated molecular patterns (DAMPs)**

Also known as damage-associated molecular pattern molecules; molecules, often nuclear or cytosolic proteins like HMGB1, that can initiate and perpetuate immune response in the non-infectious inflammatory response.

**Endotoxemia**

The presence of endotoxins in the blood, which may cause haemorrhages, necrosis of the kidneys and shock.

**Hyperdynamic phase**

Early phase of sepsis characterized by increased cardiac output, tachycardia, fever, leukocytosis (neutrophilia).

**Hypodynamic phase**

Late phase of sepsis characterized by reduced cardiac output, bradycardia, hypotension, hypothermia, leukopenia.

**Immunomodulators**

Components that can either enhance or suppress immune responses.

**Immunostimulants**

Agents that will bring about increased immune responsiveness, especially in situations in which immune defenses (whether innate and/or adaptive) have been degraded.

**Multiorgan failure**

Also known as multiple organ dysfunction syndrome or multisystem organ failure; the presence of altered organ function in a patient who is acutely ill and in whom homeostasis cannot be maintained without intervention.

**Pathogen-associated molecular patterns (PAMPs)**

Pathogen-derived molecules recognized by cells of the innate immune response that initiate and perpetuate the infectious inflammatory response.

**Reactive nitrogen species (RNS)**

Family of antimicrobial molecules derived from nitric oxide (^**.**^NO) and superoxide (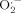) produced via the enzymatic activity of inducible nitric oxide synthase 2 (NOS2) and NADPH oxidase, respectively.

**Reactive oxygen species (ROS)**

Oxygen radicals that are mainly produced by the mitochondrial respiratory chain. In excess, they can cause intracellular and mitochondrial damage, which promotes cell death.

**Sepsis**

An illness in which the body has a severe response to bacteria, other pathogens or sterile inflammation. Stages of clinical sepsis are sepsis (accompanied by a systemic response to infection including fever, neutrophilia, tachycardia, increased breathing rate, etc.); severe sepsis and systemic inflammatory response syndrome (SIRS); multiorgan failure (MOF) involving lungs, liver, kidneys, heart; septic shock.

**Septic shock**

A state of acute circulatory failure characterized by persistent arterial hypotension despite adequate fluid resuscitation or by tissue hypoperfusion unexplained by other causes.

**Severe sepsis**

Sepsis complicated by end-organ dysfunction, as signalled by altered mental status, an episode of hypotension, elevated creatinine concentration or evidence of disseminated intravascular coagulopathy.

**‘Sterile’ inflammation**

Inflammation as a result of trauma, ischemia-reperfusion injury or chemically induced injury that typically occurs in the absence of any microorganisms.

**Systemic inflammatory response syndrome (SIRS)**

The clinical manifestations that result from the systemic response to infection. These include hyper- or hypothermia, elevated heart rate, elevated respiratory rate or decreased arterial carbon dioxide tension, abnormal white blood cell count.

Animals in the early stages of sepsis often present with a hyperdynamic phase [increased cardiac output, tachycardia, fever, leukocytosis (neutrophilia)], followed, as sepsis progresses, by a hypodynamic phase (reduced cardiac output, bradycardia, hypotension, hypothermia, leukopenia, etc.). Several similar features occur in septic humans, but clinical interventions such as antibiotic therapy and fluid resuscitation make the sequence of events less definitive than those found in animal models of sepsis. The development of MOF is associated with dysfunction of cardiac, renal, hepatic and respiratory organs but also involving the peripheral vasculature. CNS dysfunction (hallucinations, somnolence, confusion, cognitive defects, etc.) may also occur (Hermans et al, 2008; Vincent et al, 2002; Winters et al, 2010). The development of MOF may be related to hypoperfusion of organs due to falling blood pressure. Whether there is a sequential linkage between failing organs is not known. Clinically aggressive attempts are made to treat what has triggered the septic response. Simultaneously, supportive measures may include blood or plasma replacement (in the case of haemorrhagic shock), as well as infusion of resuscitative electrolyte and glucose-containing fluids, and provision of ventilator and vasopressor support. Typically in humans sepsis usually runs its acute course in 4–12 days, although FDA criteria for drug efficacy require survival data at day 28. After convalescence from sepsis, a much longer period of observation (months to years) may be appropriate because of persistence or onset of complications (cognitive defects, skeletal muscle weakness, immunosuppression, etc.).

The causes of such complications are poorly understood (Adrie et al, 2007; Angus, 2011; Angus et al, 2001; Clark & Coopersmith, 2007; Cruz et al, 2009; Deitch, 2002). Another striking feature about the longer term problems is that mortality rate years later was much higher than that found in age-matched individuals who had not experienced sepsis (Bagshaw, 2008). Accordingly, sepsis presents extremely challenging clinical problems as an acute disease but also as a long-term condition (over years) with a grave prognosis due to reduced survival.

## Animal models of sepsis

Numerous models of sepsis in animals have been developed, usually employing rodents, since the use of larger animals (rabbits, sheep, pigs and subhuman primates) would require access to ICU-type facilities with around-the-clock coverage for days or weeks. The most common models of experimental sepsis include injection (local or systemic) of live bacteria, a commonly used organism being *Escherichia coli*. Other models of sepsis include extrusion of faecal contents from surgically manipulated areas of gut [usually cecum, cecal ligation and puncture (CLP)]; the colon ascendens stent peritonitis (CASP) which causes an intraperitoneal leak of faeces, leading to polymicrobial sepsis, similar to what is seen in the CLP model; instillation of live bacteria into lung (often *Pseudomonas sp*. or *Klebsiella sp*.); and injection (local or systemic) of products of bacteria (PAMPs, see Fig 1), such as endotoxin (LPS) or lipoteichoic acid. Each of these models has its advantages and disadvantages. The CLP model has been widely employed in rats and mice and induces many of the features of human sepsis and has been referred to as the ‘gold standard’ for the study of sepsis (Dejager et al, 2011). However, the CLP model has several complexities that influence outcomes: effects of age and gender; the tremendous heterogeneity of immune and inflammatory responses related to genetic strains of mice; influences of therapeutic interventions; and dramatic differences in outcomes based on technical approaches (number of cecal punctures, needle size, amount of cecum ligated; Rittirsch et al, 2009).

There are many issues dealing with the choice of an animal model and its relevance to human sepsis, such as:

1. Dramatic differences in the timing of responses (such as when cytokine/chemokine levels peak in plasma). In rodents injected with LPS, plasma mediators peak in the first several hours *versus* in CLP with mediator peaks much more slowly developing, over the first 48 h (Remick et al, 1995; Remick & Ward, 2005; Rittirsch et al, 2007). To what extent can these two models be compared is questionable as is whether either model mimics events developing in humans with sepsis.
2. While *Klebsiella sp*. or *Pseudomonas sp*.-induced pneumonia in mice produces a sepsis pattern similar to that found in humans with Gram-negative bacterial pneumonia, to what extent do the symptoms in mice reflect those found in humans with sepsis following bacterial pneumonia? Are patterns of sepsis development due to Gram-negative bacteria similar to or different from bacterial pneumonia and sepsis induced with Gram-positive bacteria (*e.g. Streptococcal sp*., *Staphylococcus aureus*)? Does sepsis induced by Gram-negative bacterial pneumonia follow the same pathophysiological pathways occurring in sepsis related to Gram-negative bacterial peritonitis?
3. Does sepsis triggered by bacterial infection follow the same pathophysiology as found in the case of ‘sterile’ inflammation (such as seen after non-penetrating polytrauma or hemorrhagic shock)?
4. Given the difficulties in consistently demonstrating the presence of LPS in plasma from septic humans (Opal, 2002), are the animal models of endotoxemia relevant to human sepsis?

Numerous reports comprehensively review models of animal sepsis and the extent to which they may or may not be relevant to human sepsis (Buras et al, 2005; Dejager et al, 2011; Doi et al, 2009; Fink, 2008; Zanotti-Cavazzoni & Goldfarb, 2009). Other reviews emphasize the constraints of animal models when extrapolating such data to sepsis in humans (Dyson & Singer, 2009; Marshall, 2008; Rittirsch et al, 2007). There are reports suggesting that human septic males have higher mortality rates (Adrie et al, 2007; Angus et al, 2001; Schroder et al, 1998; Wichmann et al, 2000), but such reports are not consistent with others (Eachempati et al, 1999). In septic mice, there is also evidence that males have worse outcomes than female mice (reviewed in Choudhry et al, 2006). In each experimental model, it is important to document these variables since they can significantly affect the results. One animal manipulation that has been little employed to date in experimental sepsis is the use of ‘humanized mice’ in which the immune and haematopoietic systems have been replaced by human cells (Devoy et al, 2012). The use of these mice, which are extremely expensive, may provide important insights that will have greater relevance to human sepsis than the traditional use of ‘knockout’ or ‘knockin’ mice.

## Controversies about sepsis as a hyperinflammatory state and implications for human clinical trials

**Figure 2:**
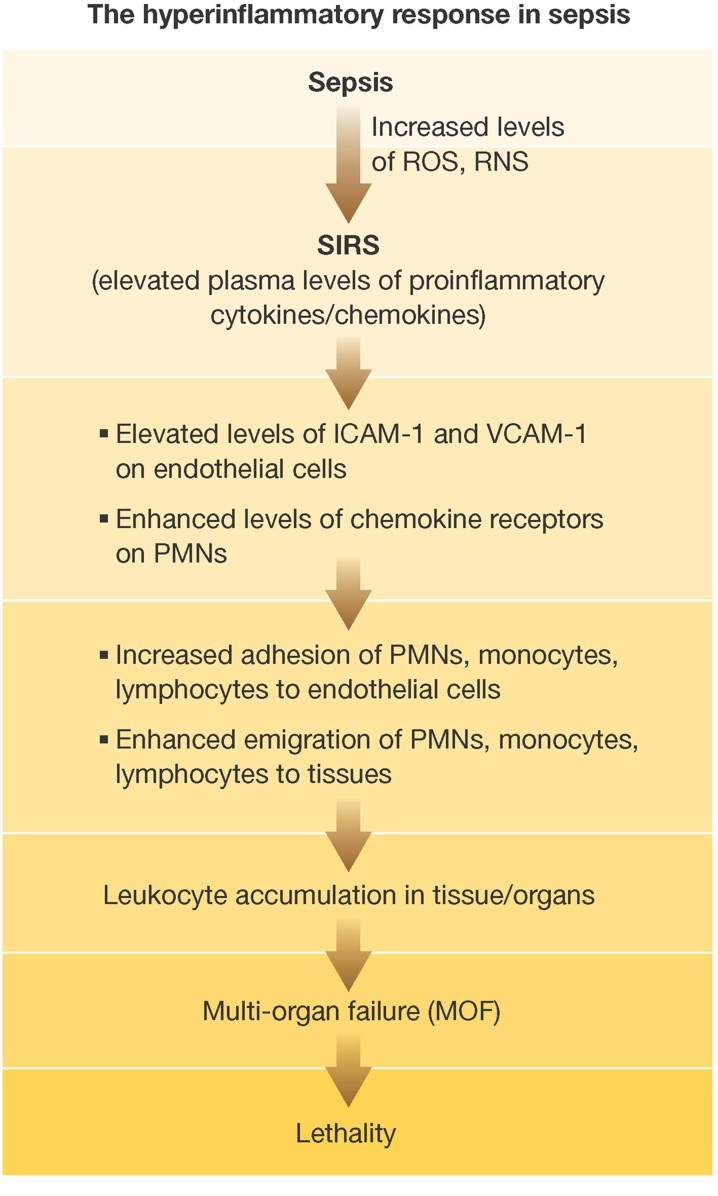
Evidence of hyperinflammatory responses developing in the setting of sepsis The early event is appearance of ROS and RNS in tissues together with numerous proinflammatory cytokines and chemokines in plasma (SIRS), accompanied by other manifestations of a robust inflammatory response (appearance of activation markers on PMNs, such as CD11b/CD18), increased levels of adhesion molecules (ICAM-1, VCAM-1) on endothelial cells, and elevated levels of chemokine receptors on PMNs, all of which implies a ‘gain of function’, resulting in a buildup in tissues of PMNs, monocytes, lymphocytes and dendritic cells in tissues. Development of activation molecules as well as chemokine receptors on leukocytes and adhesion molecules on endothelial cells would facilitate leukocyte buildup, perhaps contributing to the damage of multiple organs by facilitating the recruitment of inflammatory cells, which may be linked to MOF.

As summarized in Fig 2, there are many features in sepsis that would suggest a hyper-inflammatory state. In the setting of CLP, endothelial cells showed marked increases in surface ICAM-1 (which would favour neutrophil adherence and subsequent transmigration) and expression of VCAM-1 on endothelial cells (an adhesion molecule for lymphocytes and monocytes). CLP mice were evaluated for endothelial ICAM-1 and VCAM-1 levels using vasculature binding of ^125^I-antibodies. Progressive increases in levels of vascular ICAM-1 occurred following CLP, and such changes being found in numerous organs (Laudes et al, 2004). Concomitantly, there were also increases in vascular VCAM-1. Endothelial cells showed substantial increases in ICAM-1 mRNA during development of sepsis (Wu et al, 2002). Early in sepsis there was increased tissue content of MPO in a variety of organs. MPO is a relatively specific indicator for PMN presence (Guo & Ward, 2005). Furthermore, sepsis caused a ‘gain of function’ in neutrophils, with increased levels of β_2_ integrins (*e.g.* CD11b/CD18; which indicates activation) together with increased levels of β_1_ integrin, inferring that these cells would now be interactive with connective tissue matrix proteins (Speyer et al, 2004; Speyer & Ward, 2011). Also, blood PMNs during sepsis acquired increased expression of mRNA for CCR2 and CCR5, correlating with enhanced ligand binding and intensified *in vitro* chemotactic responses to various chemokines. Together, these data are consistent with a hyper-inflammatory state developing in rodents after CLP, the duration of which is unknown.

The cell sources for mediators of the SIRS response are chiefly derivatives of myeloid cells (*e.g.* PMNs, macrophages, monocytes, dendritic cells) but also non-myeloid cells (*e.g.* hepatocytes, alveolar epithelial cells, cardiomyocytes, etc). The downstream effects of the SIRS mediators are not clearly defined. While cytokine/chemokine interaction with receptors in various organs may lead to organ injury, there is no direct evidence to support such conclusions. The problem in precisely defining the role of these proinflammatory mediators is highlighted by the substantial overlap and functional redundancy of these mediators. In addition, there are several individual receptors that can bind with more than one ligand. For all of these reasons, the strategy in sepsis to target a single cytokine or chemokine for neutralization would not be expected to be successful. This may explain the general failure in human clinical trials when a single mediator was selected as a target for blockade, such as antibody neutralization of TNF-α, blocking of TNF-α with soluble TNF-αR, or blocking of IL-1β by IL-1 receptor antagonist (reviewed in Marshall, 2008 and Vincent et al, 2002). Perhaps blockade of TNF-α or IL-1β also removed the protective function of these mediators in innate immune defenses.

The earlier literature has suggested that sepsis in rodents is associated with a breakdown in the gut epithelial barrier, resulting in translation of intact bacteria and their products (such as LPS) into the blood stream and the gut lymphatics, causing a systemic sepsis response (Deitch, 2002). In addition, it is possible that the gut also produces factors that compromise the blood-brain barrier (Banks, 2008). Based on such information, the gut has been described as the ‘motor’ of critical illness (Clark & Coopersmith, 2007). This theory is appealing, but because sepsis can also develop in the absence of infectious agents (as in ischemia-reperfusion or haemorrhagic shock) this may limit the applications of the bacterial translocation hypothesis.

Neutralization of TNF-α was highly protective and greatly improved survival in endotoxemic mice (Beutler et al, 1985). However, in CLP, the results using a neutralizing antibody to TNF-α showed no consistent evidence of protection (Eskandari et al, 1992; Remick et al, 1995). There are substantial differences in plasma/serum levels of mediators and the time when they peak in plasma of mice during endotoxemia as compared to CLP (Deitch, 1998; Fink & Heard, 1990; Schultz & van der Poll, 2002), raising the question about whether endotoxemia and CLP follow different pathophysiological pathways.

## Neutralization or removal of plasma cytokines/chemokines in the septic state

As indicated above, development of SIRS in sepsis refers to the presence in plasma of proinflammatory mediators, such as TNF-α, IL-1β, IL-6, IL-18, IL-8 as well as MCP-1 (CCL-2), MIP-1α (CCL3), MIP-1β (CCL4), etc. Some of these mediators are chemotactic for PMNs and monocytes, while others are chemotactic for T cells. Biological responses induced by these mediators are quite pleiotropic. SIRS is also associated with elevated levels of ROS and RNS in organs as sepsis progresses to ‘severe sepsis’ and development of septic shock and multi-organ failure (Fig 2). The temporal patterns of the SIRS response, and the precise relationships between redox imbalance, development of SIRS and subsequent progression of MOF are uncertain. While limited data (see above) suggest that SIRS does not fully develop if the redox imbalance is at least partially reversed, it is still questionable as to whether SIRS can be linked to redox imbalance. It is assumed but not proven that the plasma mediators may lead to the buildup of MPO as well as ROS and RNS levels in various organs which eventually fail in sepsis. In contrast, it has been suggested that SIRS may reflect inadequate removal of the infectious agent responsible for the development of sepsis (Angus, 2011), although in the case of ‘sterile’ inflammation after polytrauma, ischemia-reperfusion injury or haemorrhagic shock, the role of an infectious agent is not likely. Regardless of the cause of SIRS and subsequent responses, bulk removal of cytokines/chemokines from plasma might provide clues as to the roles of these mediators. Haemodialysis has not been shown to be effective in removal of plasma cytokines/chemokines (Cole et al, 2002; Sieberth & Kierdorf, 1999). In 2002, *ex vivo* removal using an extracorporeal circuit was employed together with large-pore haemofiltration and sorbent adsorption. Fresh human blood had been activated with LPS. This blood was then recirculated through the device for 6 h. Over the first few hours, significant reductions in IL-1, IL-6, IL-8 and TNF-α occurred (Cole et al, 2002). Haemofiltration devices have been employed for several decades, being used chiefly in patients with acute renal failure (ARF). In the earlier models of continuous haemofiltration, reductions in plasma TNF-α were transient (Sieberth & Kierdorf, 1999). Haemofiltration through a sorbent (which removes plasma peptides/proteins) resulted in reductions of IL-1, IL-6, IL-10, IL-18, TNF-α and other mediators (Winchester et al, 2003). Apheresis (use of columns to remove selective components of blood) employing immobilized polymyxin B fibre columns was able to remove LPS from human blood (Shimizu et al, 2006), but for the reasons listed above, this may not be an especially effective strategy for the treatment of many humans with sepsis who have Gram-positive sepsis. In multicentre clinical trials in Italy involving patients with severe sepsis or septic shock associated with intra-abdominal Gram-negative infections, polymyxin B haemoperfusion appeared to significantly improve haemodynamics and organ dysfunction (Cruz et al, 2009). Recently, porous carbon particles that were blood compatible were shown to rapidly remove TNF-α, IL-6 and IL-1β from plasma (Yachamaneni et al, 2010). Continuous haemofiltration has been employed using a polymethylmethacrylate membrane haemofilter in 43 patients with septic shock. Plasma levels of IL-6 were reduced and haemodynamic indices were improved, together with increased urine output and reduced levels of blood lactate (Nakada et al, 2008). Obviously, further clinical studies are needed together with determinations of precisely what mediators were removed and for how long. Concerns include the highly invasive nature of many of these techniques. The ultimate, critical question is: to what extent will mediator removal from septic human blood reduce the progression to septic shock and multi-organ failure?

Finally, there has been consideration for the use of anti-inflammatory corticosteroids/glucocorticoids in the setting of sepsis. While early clinical studies suggested beneficial effects of steroids in sepsis (Schumer, 1976), subsequent studies failed to show any benefit with high doses of steroids. Recent studies have suggested that low doses of hydrocortisone may have some beneficial effects in sepsis (reviewed in Vincent et al, 2002) but in general there has been limited enthusiasm for the use of steroids in septic humans. In addition, a recent report cautions that systemic corticosteroid treatment should be very cautiously employed in critically ill patients in ICUs because of greatly increased risk of death due to bacterial pneumonias (Ranzani et al, 2012).

## Use of immunostimulants in sepsis

The term ‘immunostimulants’ connotes agents that will bring about increased immune responsiveness, especially in situations in which immune defenses (whether innate and/or adaptive) have been degraded. Immunomodulators are components that can either enhance or suppress immune responses. Immunostimulants have been used in patients with progressive cancer in attempts to boost the immune system and suppress tumour progression. Although IFN-γ and IL-2 have been used to enhance immune responsiveness, their use has been greatly limited due to intense toxicity at doses employed clinically.

Development of immunosuppression early in the onset of sepsis in humans is well established. Most patients diagnosed with sepsis (only 50% having a confirmed presence of an infectious organism by the time the diagnosis of sepsis has been made) are already severely lymphopenic. Recent studies indicate that apoptosis of T and B cells is often well advanced at the time of admission to the ICU (Boomer et al, 2011; Hotchkiss & Karl, 2003; Russell, 2006). The pathways of apoptosis in sepsis may involve activation of the extrinsic (TNF-α/TNFR and Fas/FasL) and/or intrinsic (mitochondrial) pathways, the final steps being activation of caspases that cause apoptosis of T and B cells. The studies of the Hotchkiss group showed that synthetic, broad spectrum inhibitors of apoptosis (such as Z-VAD), if given before onset of CLP in mice, would reduce caspase activation and cause diminished intensity of lymphoid cell apoptosis, resulting in improved survival after CLP (Hotchkiss et al, 1999). It was also shown that use of Z-VAD-FMK, a broad spectrum caspase inhibitor, reduced plasma levels of HMGB1, a mediator known to be harmful in the setting of endotoxemia. Precisely how Z-VAD-FMK caused reduction of HMGB1 is not clear. However, commencement of the anti-apoptotic therapy after the onset of CLP had marginal effects.

Because of these difficulties, there has been interest in the use of immunostimulants in septic humans, with the hope that they might at least partially restore immune competence through regeneration of the lymphoid system decimated by extensive apoptosis of T and B cells (Unsinger et al, 2010). IL-7 is a potent anti-apoptotic cytokine that promotes lymphocyte survival and is a growth-promoting factor for both T and B cells. Its toxicity is very limited. When given 90 min after induction of CLP in mice, IL-7 improved survival and reduced apoptosis of CD4 and CD8 positive cells, restored IFN-γ production, and improved immune effector cell recruitment. This treatment also restored the ability of CLP mice to express delayed-type hypersensitivity responses in skin. Increased expression of the major anti-apoptotic protein, Bcl-2, in lymphocytes also occurred. IL-7 induces proliferation of naïve CD4 and CD8 T cells (Fry & Mackall, 2005; Geiselhart et al, 2001) and has been used in clinical trials in cancer patients in an attempt to bolster immune defenses (Sportes et al, 2008). Clinical trials using IL-7 in humans with sepsis have not yet commenced, but such interventions may hold promise on the basis of results in CLP mice and increases in blood CD4^+^ and CD8^+^ lymphocytes in HIV patients (Banks, 2008).

Another potential immunostimulant for septic patients is IL-15. This cytokine has biochemical similarity to IL-2. It is produced by macrophages and induces proliferation of NK cells and other cells of the innate immune system. It regulates T-cell activation and proliferation and induces Bcl-2 to render T cells resistant to apoptosis. In experimental animals, over-expression of IL-15 protected against *E. coli* induced shock, perhaps because levels of TNF-α were reduced (Hiromatsu et al, 2003). Based on these features, IL-15 may also be a candidate for reversal of immunosuppression in sepsis.

The broad area of immunostimulants, including for use in sepsis, has been recently reviewed (Webster & Galley, 2009). Many of the strategies (glucocorticoids, sedatives, opioids and corticosteroids) have already been used in sepsis and appear to have limited beneficial effects. Other interventions provide evidence for enhanced immune responses including glucans (Gertsch et al, 2011; Vetvicka, 2011) and muramyl peptides (O'Reilly & Zak, 1992). While most of these immunostimulants have been used to treat patients with malignant tumours, there is little information on whether they would be useful in sepsis to rescue the immune system or reverse the state of immunosuppression.

## Neutralization of C5a and C5a receptors in sepsis

**Figure 3:**
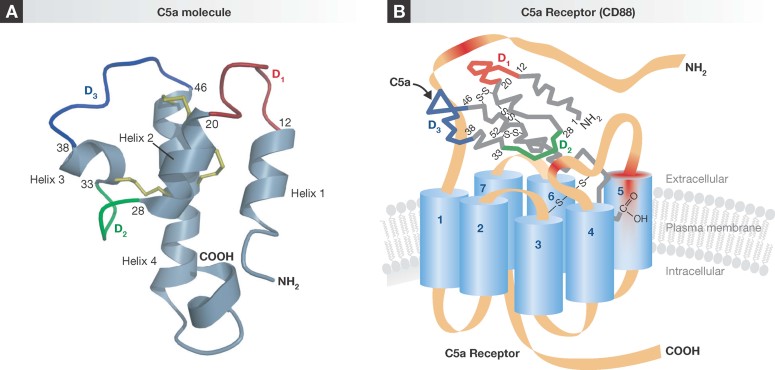
Molecular models of C5a and C5aR in which its ligand, C5a, is also shown C5a is a rather oxidant-resistant glycosylated peptide with antiparallel helical structures.The interaction of C5a with C5aR (CD88) triggers series of responses initiated by interaction of the cytoplasmic tail of C5aR with G-proteins, followed by engagement and activation of the signalling molecules, RAS, RAF-1, MEK1/2 and ERK1/2. An important response is activation of NADPH oxidase (NOX2) which is linked to the translocation of cytosolic subunits of NOX2 to the plasma membrane where NOX2 is assembled, facilitating electron transport and production of superoxide anion (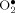) and generation of H_2_O_2_, which accounts for the major bactericidal activity of activated PMNs. The presence of activated PMNs would result in inflammatory injury of organs.

As indicated above, in the 1980s it was found that endotoxemic mice responded with brief, high levels of TNF-α in plasma and that systemic neutralization of TNF-α significantly improved survival (Beutler et al, 1985). In baboons that had been infused with live *E. coli*, a similar strategy for neutralization of TNF-α produced beneficial effects and improved survival (Tracey et al, 1987). When monkeys that developed septic shock after infusion of live *E. coli* were pre-treated with rabbit polyclonal IgG that neutralized C5a, there were suggestions of benefit (based on clinical findings, biochemical and metabolic parameters and mortality), but the small number of animals precluded the drawing of statistically significant conclusions (Stevens et al, 1986). Experimental evidence (chiefly from our own research laboratories) has provided evidence for a key role of C5a and its receptors (C5aR, C5L2) in the progression of experimental sepsis (CLP) in rodents (reviewed in Ward, 2010). In sepsis involving rodents or humans, there is robust activation of complement and the appearance in plasma of the anaphylatoxins, C3a and C5a. The latter binds with high affinity to its receptors, C5aR and C5L2, especially on phagocytes (neutrophils, monocytes and macrophages) but also on a variety of non-myeloid derived cells [such as endothelial cells and cardiomyocytes (Gerard et al, 2005; Monk et al, 2007; Okinaga et al, 2003)]. The structures of C5a and the C5a receptor (C5aR, CD88) are shown in Fig 3. Human C5a is a 74 amino acid glycoprotein (while rat C5a has 77 residues) with complex anti-parallel helical structures (Fig 3A). C5a is released from the α chain of the parent molecule, C5, following activation of complement, whether by the classical, alternative or lectin pathways. PMNs have high numbers of binding sites for C5a (circa 60,000 per cell), while monocytes, macrophages and endothelial cells have far fewer binding sites. C5aR is a 45 kDa protein, rhodopsin-type transmembrane spanning structure that is coupled to G proteins in the cytosol (Fig 3B). Activation of C5aR by C5a is followed by activation of the following signalling proteins: RAS, RAF-1, MEK1/2 and ERK1/2, unleashing a series of functional responses, including activation of NADPH oxidase (NOX2). Details are provided elsewhere (Ward, 2004).

In PMNs, C5a triggers G protein binding to the C terminal region of C5aR, resulting in a cascade of signalling events (described above), with biological responses such as priming for accentuated cell responses to a second stimulus (*e.g.* LPS, Wrann et al, 2007). Responses to C5a via C5aR also include intracellular Ca^2+^ transients, secretion of cytoplasmic granule contents, activation of NADPH oxidase which results in production of ROS [*e.g.* superoxide anion (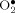), H_2_O_2_, etc.], chemotactic responses and a variety of other functional responses (Wrann et al, 2007), which represent innate immune protective responses in the setting of infectious agents or after organ injury (*e.g.* trauma, ischemia/reperfusion injury, etc.). In contrast, the second C5a receptor, C5L2, contains substitutions in C terminal amino acids such that there is no interaction with G proteins (Monk et al, 2007), with no [Ca^2+^]i transients when these receptors interact with C5a or C5a des arg. It is still controversial as to whether C5L2 receptor activation after contact with ligand induces signalling responses that result in activation of MEK1/2 and ERK1/2 (Chen et al, 2007) or whether C5L2 simply functions as a ‘default’ receptor. There is controversy regarding whether C5L2 in the ‘resting’ PMN is expressed on the cell surface or only within the cytosol (Monk et al, 2007). To date, understanding C5L2 function is uncertain.

Neutralization (with antibody) or genetic absence of C5aR or C5L2 reduces the intensity of CLP or endotoxemia in mice as reflected in survival curves (Chen et al, 2007; Guo & Ward, 2005; Rittirsch et al, 2008). When both C5a receptors were unavailable, survival rates in CLP mice were improved and plasma levels of cytokines/chemokines were significantly reduced, suggesting that activation of both C5a receptors occurs in CLP and results in harmful outcomes (Rittirsch et al, 2008). Blockade (with neutralizing antibodies) or genetic absence of C5aR or C5L2 improved survival in CLP or endotoxemic mice (Chen et al, 2007; Guo & Ward, 2005; Rittirsch et al, 2008).

With respect to the ability of C5a neutralizing antibodies to protect CLP rats from lethality, three different regions of rat C5a were selected as targets. These included the N-terminal (N) region (residues 1–16), the middle (M) region of C5a (residues 17–36) and the C terminal region (residues 58–77). Rabbit polyclonal IgG antibodies obtained were affinity-purified as antigen (C5a peptide)-specific IgGs. Antibody to the N region produced intense bands on Western blot analysis but had no protective effects in CLP rats, whereas antibodies to the M and C terminal regions of rat C5a were highly protective (Huber-Lang et al, 2001; Rittirsch et al, 2008). It was also shown that delayed administration of these antibodies, up to 12 h after CLP, still provided protective effects. What is the most desirable target for treatment in sepsis (C5a, C5aR or C5L2)? Since the two C5a receptors are also expressed on a variety of non-myeloid cells/tissues, it is unclear if antibodies to these receptors might have unintended consequences such as agonist functions, raising the question about the duration of C5a receptor blockade *in vivo*. There are small molecular weight molecules with antagonistic effects on C5a receptors, but their pharmacokinetics characteristics are inadequately known, and they have only been applied in humans in very limited circumstances (rheumatoid arthritis) without clinical efficacy (Woodruff et al, 2011). Taken together, this information suggests that targeting C5a rather than C5a receptors may be the desirable option for treatment of humans with sepsis.

## The future

As mentioned in the Introduction section, there is a desperate need for new, effective drugs for treatment of humans with sepsis. Based on more than 40 failed clinical trials in septic humans, it is clear that extrapolation of data obtained from septic (CLP) or endotoxemic rodents does not necessarily provide reliable predictions for septic humans. One possibility is that the pathophysiology of sepsis in humans is different from events occurring in septic rodents. As indicated above, resuscitative measures in humans may alter the pathophysiological manifestations of sepsis. Most CLP or endotoxemic studies employ 25 g C57Bl/6 male mice which are 1–2 months old. This would be roughly equivalent to young humans (approximately 4 years old), while in most clinical trials the mean age of patients is 60 years. Accordingly, this may be one reason why animal data cannot be reliably extrapolated to humans. Another possibility is that clinical trial design in humans has been far less than optimal. For instance, when only about 30% of all septic patients will expire in the course of 28 days, this means that nearly 70% of patients will not show benefit with new drug interventions. Such a reality means that the number of enrollees needs to be very large (thousands) in order to achieve statistical significance for a drug that is ultimately shown to be efficacious. Another issue with clinical trial design is that better selection of septic patients (such as those entering into septic shock) might allow for much smaller clinical trials. Obviously, we do not have enough information to predict if a cohort would demonstrate beneficial effects of drug interventions, or if the onset of septic shock represents an advanced point of sepsis that is too late for rescue. Needless to say, we need much more information about the pathophysiology of sepsis and how we can most effectively intervene and at what time after the diagnosis of sepsis. Other considerations that need to be factored in relate to co-morbidities (diabetes melitis, obesity, cardiovascular disease, hypertension, etc.) that are common in septic patients (usually approximately 60 years of age).

**Pending issues**

**Animal models:** As emphasized in the text, rodent models of sepsis have often employed the CLP model. The outcomes are vitally dependent on technical details (number of cecal punctures, needle gauge, amount of cecum ligated). The critical question is the relevance of findings in the CLP model to events developing in human sepsis. No consensus has been reached on this matter. Interventions that were protective in CLP mice were not beneficial in septic humans. One possibility that might yield useful data would be using the CLP model in ‘humanized mice’ that have human myelopoietic and lymphoid cells, use of which might provide data more relevant to septic humans.

Another issue is the relevance of the endotoxemia mouse model to humans with sepsis. There are many reasons to question the relevance of the endotoxemia mouse model to humans with sepsis, as described in the text. While endotoxemia in both mice and in humans unleashes a cascade of similar responses (complement activation, neutropenia followed by neutrophilia, activation of the clotting cascade, etc.), such outcomes may be relevant to septic humans with Gram-negative infections but there are serious questions as to whether this information applies to humans with Gram-positive sepsis (*e.g. Streptococcal pneumoniae*, *Staphylococcus aureus*).

**Future directions:** On the basis of the failures in more than 40 clinical trials in humans with sepsis, a major problem is that the current lethality rate is 25–30%, indicating that 70% of patients will not likely show benefit from new drugs. As indicated in the text, there may be strategies for patient recruitment that would permit doing much smaller clinical trials.
